# Rare finding of *Corynebacterium bovis* in a facial surgical wound

**DOI:** 10.1093/skinhd/vzae005

**Published:** 2025-01-22

**Authors:** Sach Thakker, Micah Belzberg, Elise Ng

**Affiliations:** Department of Dermatology, Johns Hopkins University School of Medicine, Baltimore, MD, USA; Department of Dermatology, Johns Hopkins University School of Medicine, Baltimore, MD, USA; Department of Dermatology, Cleveland Clinic, Cleveland, OH, USA; Department of Dermatology, Johns Hopkins University School of Medicine, Baltimore, MD, USA

**Figure 1:**
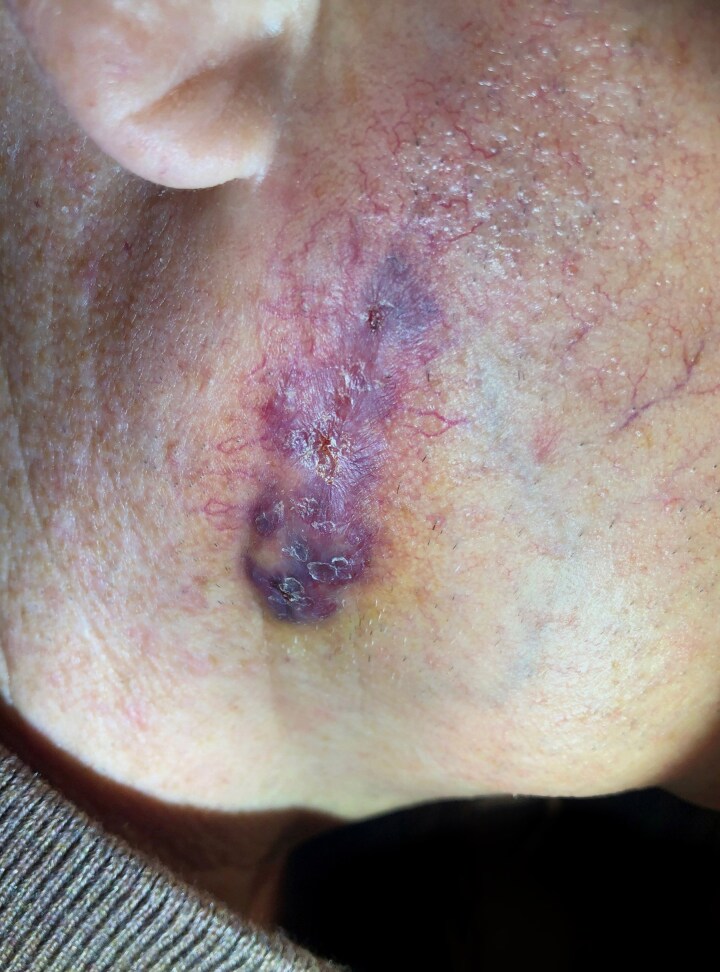
Clinical image of the postoperative site 1 month after surgery. An atypical appearing asymmetric violaceous and erythematous thinly desquamating slightly indurated plaque without fluctuance developed 2 weeks after Mohs surgery and 1 week after superficial suture removal. Two punch biopsies were performed at the inferior edge of the incision for haematoxylin and eosin staining and tissue culture.

**Figure 2:**
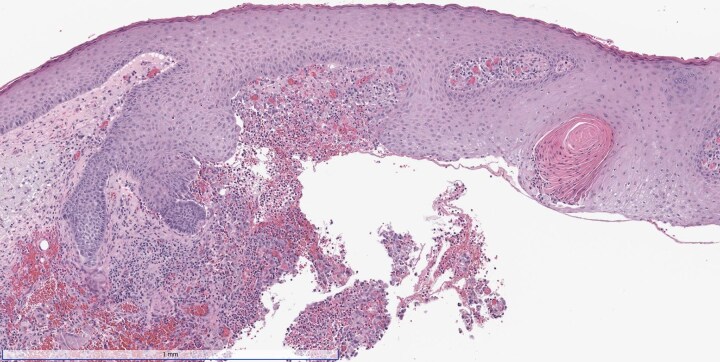
Histological stain. A 4-mm punch biopsy of the wound revealed a granulomatous and neutrophilic infiltrate with interstitial haemorrhage. Gram stain highlighted clusters of Gram-positive cocci and coccobacilli within the infiltrate.

Dear Editor, A man in his 50s developed atypical operative site skin changes 2 weeks after Mohs surgery for an invasive squamous cell carcinoma on his right cheek [American Joint Committee on Cancer (AJCC) stage T1, Brigham and Women’s Hospital (BWH) stage T1]. The growth was cleared in a single stage and closed in a layered fashion with polyglactin 910 and polypropylene sutures. His medical history included a liver transplant for which he was taking long-term tacrolimus for immunosuppression; and a deep venous thrombosis for which he was taking apixaban. Initially, the wound healed well without signs of infection. However, 2 weeks postsurgery the patient experienced progressive redness, dull pain and swelling near the suture line. Oral doxycycline provided slight improvement, but symptoms worsened, prompting re-evaluation. Physical examination revealed a violaceous, erythematous plaque with induration and patterned purpuric plaques at the suture positions (Figure 1). Punch biopsy showed a granulomatous and neutrophilic infiltrate, while Gram stain highlighted clusters of Gram-positive cocci and coccobacilli (Figure 2). Wound swab was deferred in the absence of purulent drainage or fluctuance. An additional punch biopsy for culture grew *Corynebacterium bovis* as identified using matrix-assisted laser desorption–­ionization time-of-flight mass spectrometry. Susceptibility testing revealed sensitivities to erythromycin, gentamicin, penicillin and vancomycin. Our institution’s infectious disease department chose a 7-day course of amoxicillin 500 mg three times daily, based on the results of the sensitivity testing, in respect of the ‘antibiotic ladder’ and in accordance with the literature.^1–3^ The patient subsequently experienced complete symptom resolution. The infection resolved with antibiotic treatment alone, avoiding suture removal and surgical revision.

*Corynebacterium bovis* is a facultatively anaerobic, catalase-positive, Gram-positive bacterium, commonly associated with bovine mastitis and dermatitis in immunocompromised mice. Human infections are rare, with only 18 reported cases, primarily at ocular, prosthetic joint or neurosurgical sites.^1,2^ In this case, the source of infection was unclear as the patient denied contact with farm animals or rodents. *Corynebacterium bovis* has been isolated from Merino sheep in Argentina, and following surgery the patient wore a new Merino sweater.^3^ There are no established guidelines for *C. bovis* infections in humans, although penicillin has shown effectiveness.^1–3^ However, the literature notes increasing instances of biofilm-forming and multidrug-resistant strains of *Corynebacterium*, including *C. bovis*, emphasizing the need for targeted antimicrobial treatment.^4^ Unlike other *Corynebacterium* species, *C. bovis* is not among normal human flora, and its presence in deeper tissue cultures should not be dismissed as contamination.^5^ Additionally, polyglactin 910 sutures often induce granulomatous reactions and are more susceptible to biofilm formation compared with non-braided sutures, suggesting avoidance of these suture types in patients at increased risk of infection, such as those on long-term immunosuppression.^6,7^ Evidence supporting antibiotic prophylaxis for immunosuppressed patients undergoing dermatological procedures is mixed and it is therefore not routinely performed at our institution.^8^

Funding sources: This research received no specific grant from any funding agency in the public, commercial or not-for-profit sectors.

Data availability: The data underlying this article will be shared on reasonable request to the corresponding author.

Ethics statement: Not applicable.

Patient consent: Written patient consent for publication was obtained.
